# Supplementary material for: Genomic dissection of Klebsiella pneumoniae infections in hospital patients reveals insights into an opportunistic pathogen
Source: Nat Commun. 2022 May 31;13:3017. doi: 10.1038/s41467-022-30717-6 (PMC9156735; doi:10.1038/s41467-022-30717-6)
Supplement: Supplementary file 1 — Supplementary Information [file 41467_2022_30717_MOESM1_ESM.pdf]

Supplementary Figures and Tables

accompanying the article

Genomic dissection of *Klebsiella pneumoniae* infections in hospital patients reveals insights into an opportunistic pathogen

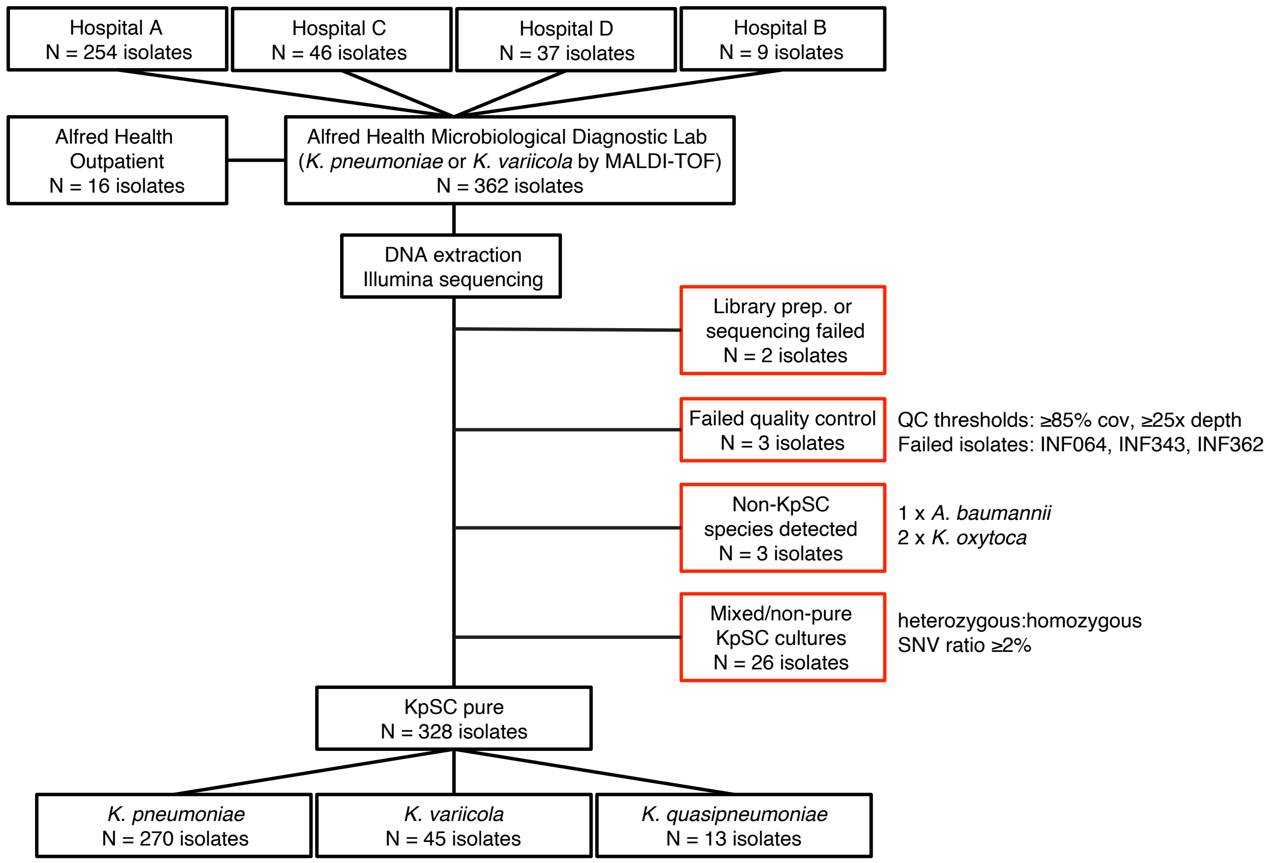

Supplementary Figure 1: Flowchart for collection and sequencing of clinical isolates.

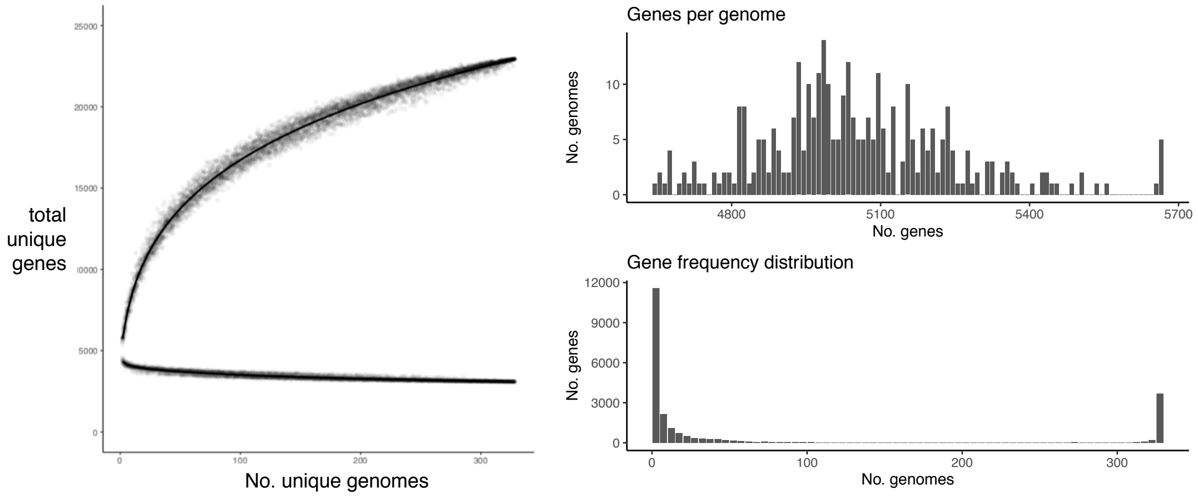

Supplementary Figure 2: Pan genome.
Data used for this analysis was all KpSC genomes. (a) Upper curve shows the pan genome, i.e. total unique genes encountered (y-axis) in a sample of *n* genomes (x-axis). Lower curve shows the core genome, i.e. total genes shared by all genomes (y-axis), in a sample of *n* genomes (x-axis). Points correspond to 20 random samples of *n* genomes, for *n*=1, …, 328; curves are Loess smoothed curves plotted through these points using the geom_smooth() function in R, with the formula 'y~log(x)'. (b) Distribution of total number of genes per genome. (c) Distribution of gene frequencies across the set of 328 genomes.

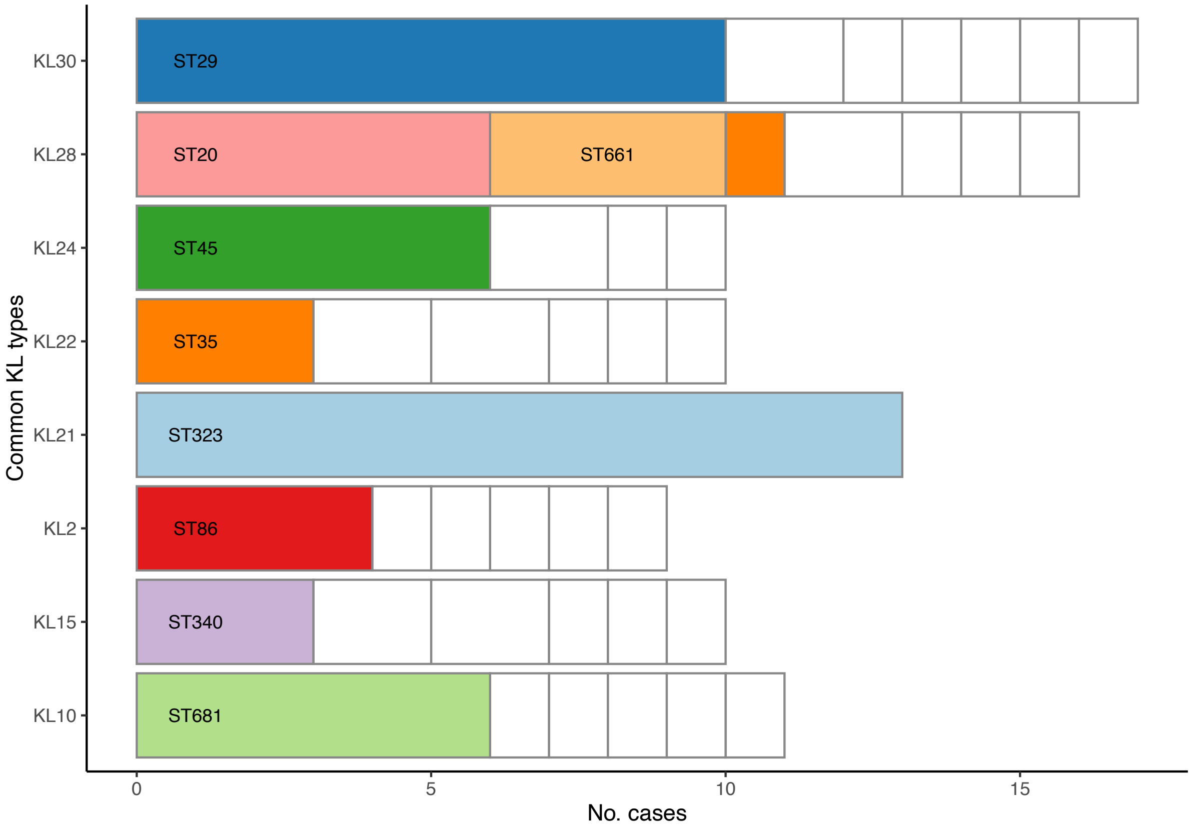

Supplementary Figure 3: Eight most common K locus types.
Data used for this analysis was the first isolate per unique infection episode, for 294 infection episodes. Stacked bars represent different K locus (KL) types and are stratified by ST; common STs are coloured and labelled; rare STs are coloured white.

Supplementary Table 1: Capsule (K) biosynthesis loci identified in clinical isolates

KL	No. infections	No. STs	*man* operon	*rml* operon
KL30	17	7	yes	no
KL28	16	7	yes	no
KL21	13	1	yes	no
KL10	11	6	yes	no
KL15	10	6	no	no
KL22	10	6	no	no
KL24	10	4	yes	no
KL2	9	6	yes	no
KL14	8	4	yes	yes
KL25	8	5	no	no
KL63	8	5	yes*	no
KL39	6	2	yes	no
KL47	6	6	no	yes
KL54	6	5	yes*	no
KL55	6	5	no	yes
KL64	6	4	yes	yes
KL102	5	3	no	no
KL142	5	5	no	yes
KL16	5	4	yes*	no
KL118	4	1	no	yes
KL38	4	4	no	no
KL53	4	3	yes	yes
KL57	4	3	yes	no
KL116	3	1	yes	no
KL13	3	3	yes	no
KL20	3	3	yes	no
KL3	3	3	yes	no
KL52	3	2	no	yes
KL60	3	3	yes	no
KL7	3	2	yes	no
KL9	3	3	no	yes
KL103	2	2	no	yes
KL105	2	2	no	yes
KL109	2	1	yes	yes
KL114	2	2	yes	no
KL122	2	2	yes	no
KL124	2	1	no	yes
KL125	2	2	no	no
KL143	2	2	yes	no
KL169	2	2	yes	yes
KL170	2	1	no	yes
KL36	2	2	no	yes
KL46	2	2	yes	no
KL48	2	2	no	yes
KL51	2	2	no	no
KL58	2	2	yes*	no
KL62	2	2	yes	no
KL71	2	2	no	yes
KL8	2	2	no	no
KL1	1	1	yes*	no
KL101	1	1	no	yes
KL107	1	1	no	yes
KL108	1	1	yes	no
KL11	1	1	no	no
KL111	1	1	no	no

KL	No. infections	No. STs	*man* operon	*rml* operon
KL112	1	1	yes	no
KL113	1	1	yes	no
KL119	1	1	yes	no
KL12	1	1	no	yes
KL120	1	1	no	yes
KL123	1	1	no	no
KL127	1	1	no	yes
KL130	1	1	yes	yes
KL131	1	1	no	yes
KL132	1	1	yes	no
KL133	1	1	yes	no
KL134	1	1	no	no
KL137	1	1	yes	yes
KL139	1	1	yes	no
KL140	1	1	yes	no
KL141	1	1	yes	yes
KL144	1	1	yes	yes
KL146	1	1	yes	no
KL153	1	1	yes	no
KL158	1	1	no	yes
KL166	1	1	yes	yes
KL167	1	1	yes	no
KL168	1	1	yes	yes
KL17	1	1	no	yes
KL18	1	1	no	yes
KL19	1	1	no	yes
KL23	1	1	no	yes
KL34	1	1	no	yes
KL35	1	1	yes	no
KL37	1	1	no	no
KL4	1	1	no^	no
KL42	1	1	yes	no
KL45	1	1	no	yes
KL5	1	1	yes	no
KL61	1	1	yes	no
KL67	1	1	yes	yes
unknown	11	11	-	-

Supplementary Table 1: Capsule (K) biosynthesis loci identified in clinical isolates. 'No. STs' indicates the number of unique multi-locus sequence types (STs) each K locus was identified in. Presence of the mannose (*man*) or rhamnose (*rml*) operons are indicated. For KL with known structures (KL1-KL82): presence of *rml* is perfectly correlated with presence of rhamnose in the expressed capsular polysaccharide (CPS); * indicates KL is *man*+ but CPS lacks mannose; ^ indicates KL is *man*- but CPS contains mannose. Sugar structures for CPS encoded by KL100-KL170 are unknown.

**Species**	**ST**	**No. Patients**	**AMR**	***ybt***	**K locus**	**O type**
Kp	ST29	9	ESBL, MDR	-	$KL30^{m}$	O1
Kv	ST681	6	susceptible*	-	$KL10^{m}$	O2afg
Kp	ST323	4	ESBL, MDR	-	$KL21^{m}$	O3b
Kp	ST323	3	ESBL, MDR	-	$KL21^{m}$	O3b
Kp	ST231	3	CP, ESBL, MDR	*ybt* 15	$KL64^{m,r}$	O1
Kp	ST491	3	ESBL, MDR	-	$KL118^{r}$	OL101
Kp	ST340	3	ESBL, MDR	*ybt* 16	KL15	O4
Kp	ST2370	2	ESBL, MDR	*ybt* 4	KL15	O4
Kqs	ST5872	2	ESBL, MDR	-	$KL7^{m}$	O12
Kp	ST45	2	susceptible	*ybt* 10	$KL24^{m}$	O2a
Kqs	ST1548	2	susceptible	-	KL170	O3/O3a
Kqs	ST480	2	susceptible	-	$KL52^{r}$	OL103

Supplementary Table 2: Features of probable nosocomial transmission clusters.
Kp, K. pneumoniae; Kv, K. variicola; Kqs, K. quasipneumoniae subsp. similipneumoniae. ST, sequence type. Patients, number of patients involved in the putative cluster. AMR, indicates presence of antimicrobial resistance including CP (carbapenemase producing), ESBL (extended spectrum beta-lactamase producing), MDR (multidrug resistant, i.e. resistant to ≥3 drug classes in addition to ampicillin). *The last ST681 isolated was ESBL and MDR. *Ybt* column indicates presence of yersiniabactin lineages; K locus column indicates the capsule biosynthesis locus present; O type column indicates the O (lipopolysaccharide) antigen predicted from genome data.

Predictors	Transmission<br>*Odds Ratio*	 *95% CI*	 *p*
ESBL+	**21.0**	**9.21 – 51.1**	**<1x10^{-11}**
Yersiniabactin	1.00	0.38 – 2.49	0.997
Patient Age (years)	**0.97**	**0.95 – 1.00**	**0.017**
Patient Sex (male)	0.89	0.37 – 2.07	0.779
Onset day ≥3	**2.64**	**1.12 – 6.42**	**0.028**
man+ KL	0.52	0.17 – 1.46	0.230
man+ OL	1.22	0.49 – 3.17	0.673

Supplementary Table 3: Logistic regression model for transmission.
Results shown are for a multivariable logistic regression model of 294 unique infections, with all variables included as predictors (95% CI = 95% confidence intervals for odds ratios). All variables were coded as binary except for age, which is continuous and expressed in years. Unadjusted p-values are shown, those with p<0.05 are bolded.

Predictors	(a) Community Acquired		
	Odds Ratio	*95% CI*	*p*
Species *K. pneumoniae*	0.83	0.31 – 2.40	0.712
ESBL+	0.55	0.15 – 1.50	0.287
Ybt+	2.03	0.89 – 4.57	0.088
Iuc+	0.58	0.03 – 3.78	0.626
Patient Age (years)	1.00	0.98 – 1.02	0.930
Patient Sex (male)	0.72	0.34 – 1.47	0.371
rml+ KL	0.67	0.26 – 1.58	0.378
man+ KL	1.27	0.60 – 2.78	0.545
man+ OL	1.53	0.69 – 3.32	0.284

Predictors	(b) Onset day 3+			(c) Onset day 3+ and/or recent inpatient admission		
	Odds Ratio	*95% CI*	*p*	*Odds Ratio*	*95% CI*	*p*
Species *K. pneumoniae*	1.39	0.69 – 2.86	0.365	1.04	0.52 – 2.06	0.917
ESBL+	**2.34**	**1.18 – 4.72**	**0.015**	**2.03**	**1.04 – 4.10**	**0.042**
Ybt+	0.79	0.42 – 1.47	0.465	0.77	0.42 – 1.38	0.379
Iuc+	0.31	0.02 – 2.00	0.299	0.43	0.06 – 2.10	0.336
Patient Age (years)	1.01	0.99 – 1.02	0.406	1.00	0.99 – 1.01	0.780
Patient Sex (male)	**2.20**	**1.32 – 3.71**	**0.003**	**1.72**	**1.05 – 2.82**	**0.031**
rml+ KL	**3.12**	**1.72 – 5.74**	**<0.001**	**2.05**	**1.14 – 3.75**	**0.017**
man+ KL	0.78	0.45 – 1.35	0.369	0.77	0.46 – 1.30	0.332
man+ OL	0.87	0.49 – 1.52	0.620	0.67	0.39 – 1.15	0.146

Supplementary Table 4: Logistic regression models for infection acquisition.
Results shown are for a multivariable logistic regression model of 294 unique infections, with all variables included as predictors (95% CI = 95% confidence intervals for odds ratios). Unadjusted p-values are shown. Predictors with *P*-values below 0.05 are bolded. All variables were coded as binary except for age, which is continuous and expressed in years. (a) Outcome variable = community associated (CA) infection, i.e. isolation from an outpatient or on day 0–2 of current admission as an inpatient, and with no recorded prior contact with the Alfred Health Network (either as an inpatient or outpatient) in the previous 12 months (n=38 CA vs 256 non-CA). (b) Outcome variable = nosocomial onset, defined as isolation on day 3 or later of the current inpatient admission (n=122 vs 172). (c) Outcome variable = nosocomial, defined as isolation on day 3 or later of the current inpatient admission or with recent inpatient admission (in the last month) (n=151 vs 143).
