# Supplementary material for: Genomic dissection of Klebsiella pneumoniae infections in hospital patients reveals insights into an opportunistic pathogen
Source: Nat Commun. 2022 May 31;13:3017. doi: 10.1038/s41467-022-30717-6 (PMC9156735; doi:10.1038/s41467-022-30717-6)
Supplement: Supplementary file 3 — Description of Additional Supplementary Files [file 41467_2022_30717_MOESM3_ESM.pdf]

Description of Additional Supplementary Files

File Name: Supplementary Data 1
Description: Details of all bacterial isolates and associated patients analysed in this study. Each row indicates a unique bacterial isolate, labelled with a unique sample identifier (SampleID) that is used to refer to individual isolates throughout the manuscript. Each isolate is associated with genome data, European Nucleotide Archive (ENA) accessions are given for biosample, sequencing run and bioproject for each genome. Each isolate is also associated with the patient from which it was cultured, de-identified patient codes are given in the PatientID field; note some patient IDs are associated with multiple isolates.
