# Supplementary material for: Genomic dissection of Klebsiella pneumoniae infections in hospital patients reveals insights into an opportunistic pathogen
Source: Nat Commun. 2022 May 31;13:3017. doi: 10.1038/s41467-022-30717-6 (PMC9156735; doi:10.1038/s41467-022-30717-6)
Supplement: Supplementary file 5 — Reporting Summary [file 41467_2022_30717_MOESM5_ESM.pdf]

nature portfolio

Corresponding author(s): Kathryn Holt, Claire Gorrie

Last updated by author(s): Apr 14, 2022

Reporting Summary

Nature Portfolio wishes to improve the reproducibility of the work that we publish. This form provides structure for consistency and transparency in reporting. For further information on Nature Portfolio policies, see our Editorial Policies and the Editorial Policy Checklist.

Statistics

For all statistical analyses, confirm that the following items are present in the figure legend, table legend, main text, or Methods section.

n/a	Confirmed	
☐	☒	The exact sample size (*n*) for each experimental group/condition, given as a discrete number and unit of measurement
☐	☒	A statement on whether measurements were taken from distinct samples or whether the same sample was measured repeatedly
☐	☒	The statistical test(s) used AND whether they are one- or two-sided<br>*Only common tests should be described solely by name; describe more complex techniques in the Methods section.*
☐	☒	A description of all covariates tested
☐	☒	A description of any assumptions or corrections, such as tests of normality and adjustment for multiple comparisons
☐	☒	A full description of the statistical parameters including central tendency (e.g. means) or other basic estimates (e.g. regression coefficient) AND variation (e.g. standard deviation) or associated estimates of uncertainty (e.g. confidence intervals)
☐	☒	For null hypothesis testing, the test statistic (e.g. F, t, r) with confidence intervals, effect sizes, degrees of freedom and P value noted<br>*Give P values as exact values whenever suitable.*
☒	☐	For Bayesian analysis, information on the choice of priors and Markov chain Monte Carlo settings
☒	☐	For hierarchical and complex designs, identification of the appropriate level for tests and full reporting of outcomes
☐	☒	Estimates of effect sizes (e.g. Cohen's *d*, Pearson's *r*), indicating how they were calculated

Our web collection on statistics for biologists contains articles on many of the points above.

Software and code

Policy information about availability of computer code

Data collection	Commercial code (Microsoft Excel and Access 2013) were used to collect laboratory data relating to clinical isolates.
Data analysis	Full details of all software, including versions and citations, are given in the Methods section. All software used is free and open source.<br>Read trimming: Trim Galore v0.5.0 (github.com/FelixKrueger/TrimGalore)<br>Genome assembly: Unicycler v0.4.7<br>Annotation: Prokka v1.13.3<br>Pan-genome definition: panaroo v1.1.2<br>Read mapping: RedDog pipeline v1b.10.3 (github.com/katholt/reddog), calling Bowtie2 v2.2.5 and SamTools v1.2<br>MLST, AMR and virulence typing: Kleborate v2.0.0<br>Phylogenetic tree inference: FastTree v2.1.8<br>Capsule and O antigen locus identification: Kaptive v2.0, Bandage v0.8,1<br>Hybrid detection: Python script, available at http://github.com/rrwick/Klebsiella-assembly-species<br>Statistical analyses: R v3.6.3, including packages network (v1.17.1), ggplot2 (v3.3.5), ggnetwork (v0.5.10), ggtree (v2.4.2)

For manuscripts utilizing custom algorithms or software that are central to the research but not yet described in published literature, software must be made available to editors and reviewers. We strongly encourage code deposition in a community repository (e.g. GitHub). See the Nature Portfolio guidelines for submitting code & software for further information.

Data

Policy information about availability of data

All manuscripts must include a data availability statement. This statement should provide the following information, where applicable:

- Accession codes, unique identifiers, or web links for publicly available datasets
- A description of any restrictions on data availability
- For clinical datasets or third party data, please ensure that the statement adheres to our policy

The sequence data generated in this study have been deposited in public databases as follows. Raw Illumina reads are available in the European Nucleotide Archive (ENA), under BioProject accessions PRJEB6891 and PRJNA351909. Run accessions for each genome are provided in Supplementary Data 1. Reference-quality hybrid Illumina+ONT assemblies are available in GenBank, individual genome accessions are given in Supplementary Data 1. Illumina-only assemblies were not deposited as they are considered draft assemblies, however the full set of assemblies and pan-genome data used in this study are available in FigShare under DOI 10.26180/16811344 [https://doi.org/10.26180/16811344]. The annotated phylogeny is available in the MicroReact online viewer [http://microreact.org/project/kaspahclinical].
The third-party K. pneumoniae NTUH-K2044 sequence (GenBank accession NC_012731.1) was used as a reference genome.

Field-specific reporting

Please select the one below that is the best fit for your research. If you are not sure, read the appropriate sections before making your selection.

☒ Life sciences ☐ Behavioural & social sciences ☐ Ecological, evolutionary & environmental sciences

For a reference copy of the document with all sections, see nature.com/documents/nr-reporting-summary-flat.pdf

Life sciences study design

All studies must disclose on these points even when the disclosure is negative.

Sample size	No sample size calculations were performed as this is not relevant, all clinical isolates during the study period were included.
Data exclusions	No data were excluded from reporting of clinical laboratory findings. For genomic analyses, 32 isolates yielding insufficient K. pneumoniae species complex data were excluded, as detailed in the Methods section "Species analysis and quality control of WGS data" and Figure S1 flowchart.
Replication	This is an observational study, not experimental, hence experimental replication does not apply.
Randomization	This is an observational study, not experimental, there was no randomisation into experimental groups.
Blinding	There was no blinding as this is only relevant to intervention studies and this is not an intervention study. Data and sample collection included all clinical isolates. Informatics analysis used to define groups / features from sequence data (e.g. lineage, AMR, virulence, transmission), and statistical analyses of those groups, were done by the same analysts.

Reporting for specific materials, systems and methods

We require information from authors about some types of materials, experimental systems and methods used in many studies. Here, indicate whether each material, system or method listed is relevant to your study. If you are not sure if a list item applies to your research, read the appropriate section before selecting a response.

Materials & experimental systems

n/a	Involved in the study	
☒	☐	Antibodies
☒	☐	Eukaryotic cell lines
☒	☐	Palaeontology and archaeology
☒	☐	Animals and other organisms
☐	☒	Human research participants
☒	☐	Clinical data
☒	☐	Dual use research of concern

Methods

n/a	Involved in the study	
☒	☐	ChIP-seq
☒	☐	Flow cytometry
☒	☐	MRI-based neuroimaging

Human research participants

Policy information about studies involving human research participants

Population characteristics	All patients in whom clinical infections with K. pneumoniae species complex were diagnosed were included in the study. The

Population characteristics	patients were 55% female and ranged in age from 20 to 97 years old, with median age 70 years. The median age for females was significantly higher than for males (75 vs 67, p=0.001 using Wilcoxon rank-sum test, see Figure 1d).
Recruitment	All patients in whom clinical infections with K. pneumoniae species complex were diagnosed were included in the study. The need for consent was waived by the ethics committee as the study utilised discarded diagnostic specimens and limited demographic and clinical information drawn from hospital records, by hospital staff who would normally have access to those records. As such there is no chance of self-selection bias due to recruitment as all patients were included.
Ethics oversight	Ethical approval for this project was granted by the Alfred Hospital Ethics Committee in Melbourne, Australia (Project numbers #550/12 and #526/13). A consent waiver was granted for the inclusion of limited patient data related to clinical isolates, extracted from hospital and laboratory records by hospital staff who normally have access to the data, and shared in deidentified form with research staff for analysis in this project.

Note that full information on the approval of the study protocol must also be provided in the manuscript.
